# Papuloerythroderma of Ofuji in a Young Man

**DOI:** 10.7759/cureus.36598

**Published:** 2023-03-23

**Authors:** Raj H Patel, Kiley Fagan, Padma V Chitnavis, Douglas Grider

**Affiliations:** 1 Dermatology, Edward Via College of Osteopathic Medicine, Monroe, USA; 2 Dermatology and Mohs Surgery, Virginia Tech Carilion School of Medicine, Roanoke, USA; 3 Dermatology, Pinnacle Dermatology, Charlotte, USA; 4 Pathology, Carilion Roanoke Memorial Hospital, Roanoke, USA; 5 Basic Science Education, Virginia Tech Carilion School of Medicine, Roanoke, USA

**Keywords:** gastric cancer, eosinophilia, cutaneous lymphoma, deck-chair sign, papuloerythroderma

## Abstract

Papuloerythroderma of Ofuji (PEO) is a rare skin disorder characterized by a distinctive pattern of pruritic, flat-topped, erythematous papules which coalesce into an erythroderma-like eruption with classic sparing of the skin folds. Although the pathogenesis of this condition is incompletely understood, previous reports have suggested a notable link between PEO and various forms of malignancy and immunocompromised states. Here, we report a case of a healthy young male with no comorbidities who presented with the classical features of PEO that responded well to combination therapy comprised of topical corticosteroids and phototherapy.

## Introduction

Originally reported in Japan by Ofuji et al., Papuloerythroderma of Ofuji (PEO) is an eruption of flat-topped, pruritic, erythematous coalescing papules characteristically sparing the face and intertriginous skin, producing the classic ‘deck-chair sign’, which distinguishes it from ordinary erythroderma [1,2]. PEO is commonly associated with peripheral eosinophilia, lymphopenia, and atopy. Although there is no standardized treatment protocol, previous cases have been traditionally treated with oral corticosteroids, cyclosporine, and psoralen and ultraviolet A (PUVA) [2,3]. Some cases have also responded to immunosuppressants such as methotrexate and azathioprine [4-7].

Given its rare occurrence, the underlying etiology of PEO remains unclear. Previous reports have attempted to illustrate an association between PEO and malignancy, drugs, atopy, and infection [2]. Of these associations, malignancy was most frequently associated with the setting of PEO, specifically in patients with gastric carcinoma [8,9]. However, this finding may be confounded by the fact that a majority of cases of PEO are identified and reported in Japan, in which the incidence of gastric cancer is high. There is also a possibility that PEO may be part of a paraneoplastic syndrome in patients with underlying malignancy, although this link is poorly elucidated [10]. Due to the poor understanding of its pathogenesis and histological resemblance with cutaneous T-cell lymphoma, PEO remains an underrecognized and underdiagnosed entity. We report a case of PEO in a previously healthy young male patient without any risk factors or comorbidities. He was successfully treated in an outpatient setting.

## Case presentation

A 22-year-old South Asian male presented to the dermatology clinic for evaluation of a pruritic rash on his lower back. He first noticed pruritus about nine months prior and reports that a rough and scaly rash presented shortly after this. Two months prior, he visited his primary care physician who prescribed an oral prednisone taper and triamcinolone 0.1% cream. Although this slightly helped relieve the itching, the rash persisted. He had no past medical history and denied any personal or family history of asthma, atopic dermatitis, or seasonal allergy. He had not changed any soaps, detergents, or skin care products recently and does not take any medications or over-the-counter (OTC) supplements.

**Figure 1:**
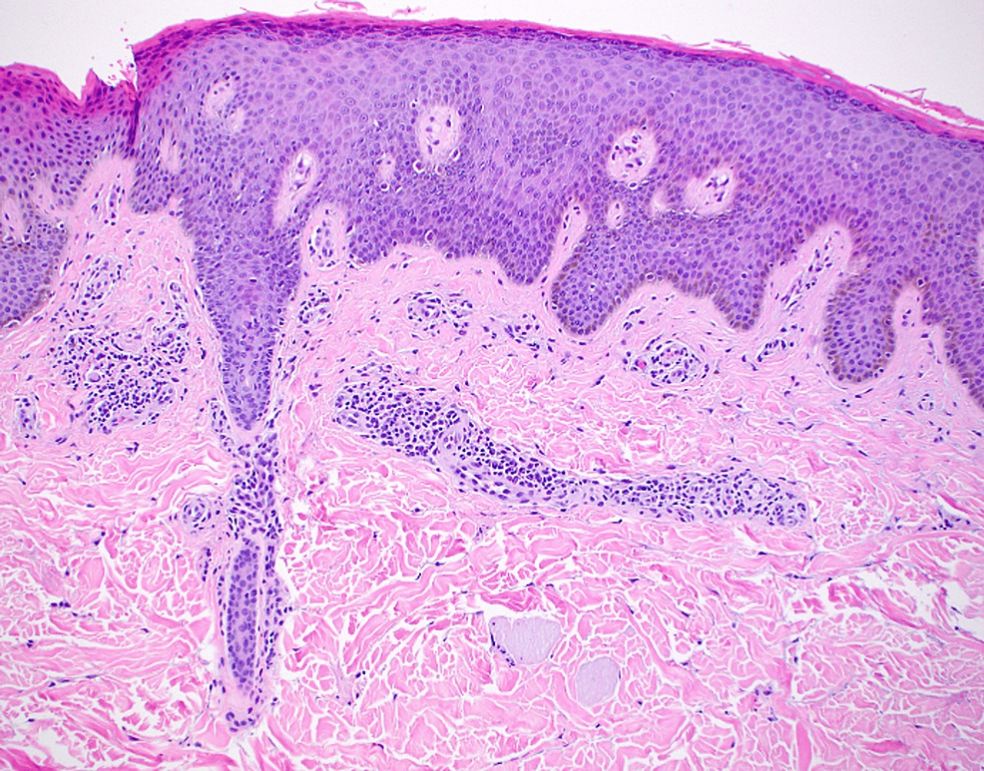
Histologic presentation obtained from lesional punch biopsy specimen. The image shows mild spongiosis with focal parakeratosis in the overlying epidermis along with perivascular lymphocytic infiltrate with eosinophils and plasma cells, S100+ dermal dendritic cells.

Dermatological examination revealed an erythroderma-like eruption formed by erythematous, flat-topped, red to brown polygonal papules with a cobblestone-like appearance which coalesced into slightly scaly, velvety, hyperpigmented plaques with notable sparing of the skin folds on the lower back. The inguinal crease, popliteal fossa, and axillary folds were spared. Focal dermal elastosis was not present. Physical examination revealed left supraclavicular lymphadenopathy. Laboratory results were largely unremarkable except for elevated liver function tests, revealing an aspartate aminotransferase (AST) of 123 U/L (normal range: 8-48 U/L) and alanine aminotransferase (ALT) of 64 U/L (normal range: 7-55 U/L). Hepatitis B and C, rapid plasma reagin (RPR), and HIV antibody titers were within normal limits. Viral capsid antigen IgG level for Epstein-Barr virus was elevated. Serum IgE level was elevated at 2941 IU/mL and complete blood count (CBC) revealed eosinophilia. Flow cytometry was also conducted to rule out cutaneous T-cell lymphoma (CTCL) of which the results were within normal limits. A punch biopsy obtained for hematoxylin and eosin staining revealed mild spongiosis with focal parakeratosis of the epidermis, and a perivascular lymphocytic infiltrate with eosinophils and plasma cells (Figure 1). Immunohistochemical staining for S100+ protein was positive for dermal dendritic cells within the perivascular inflammatory infiltrate. Based on the expected clinical, laboratory, and histopathological presentation, a diagnosis of PEO was made. 

Treatment was initiated with topical triamcinolone 0.1% cream and narrow-band UVB (nbUVB) as PUVA was not available. The patient’s skin findings and pruritus resolved after three weeks of therapy. He was also referred to gastroenterology for upper endoscopy and further workup due to the association of PEO with gastric malignancy and lymphadenopathy on physical exam, however, the patient was lost to follow-up.

## Discussion

Papuloerythroderma of Ofuji (PEO) remains a rare diagnosis that is seen to preferentially affect elderly males [3]. The proposed diagnostic criteria published in a systematic review by Torchia et al. remain a key component to accurate diagnosis, which can often be challenging due to the similar presentation by compelling differential diagnoses [8]. These diagnostic criteria include both major and minor components, in which the major components include erythroderma-like eruption formed by the coalescence of flat-topped, red-to-brown papules with a cobblestone-like appearance, sparing of skin folds and creases (creating the deck-chair sign), itchiness, histopathological exclusion of CTCL, and workup and follow-up to exclude any link with malignancies, drugs, infections, and atopy. Fulfillment of all major criteria allows for a diagnosis of primary idiopathic PEO. In addition, minor criteria, although not required for diagnosis, include an age of greater than 55 years, male sex, peripheral and/or tissue eosinophilia, increased serum IgE, and peripheral lymphopenia [8].

To date, the etiopathogenesis of PEO remains unknown. Previous case reports on PEO have demonstrated association with internal malignancy, atopy, drugs, and infection [2,11,12]. Of these, internal malignancy remains the strongest factor and some studies have also proposed that this may be tied to a paraneoplastic phenomenon, emphasizing the need for further workup for cancer. Gastric cancer in particular has been associated most frequently with PEO, underscoring the importance of endoscopy in these patients [12,13]. PEO has also been linked to CTCL, mycosis fungoides, and Sezary syndrome [14,15]. Of note, steps to diligently rule out CTCL should be undertaken as both PEO and CTCL present with similar clinical features such as a positive deck-chair sign, and can also share similar histologic findings as well [2,14]. Thus, skin biopsy alone is not sufficient to rule out CTCL, warranting the need for a comprehensive investigation including flow cytometry and T-cell receptor gene rearrangement testing [3,14]. Individuals of human T-cell leukemia virus, type 1 (HTLV-2) endemic regions should also undergo serology testing due to the frequent association with PEO in these patients [2]. Development of PEO may also be tied to dysregulation of the immune system as recent studies have illustrated a potential role of Th2 cytokines in the pathogenesis of PEO [16-18]. Treatment options remain broad and some previous reports have even demonstrated success with various immunosuppressants and monoclonal antibodies [19]. However, the most common approach involves a permutation of topical or oral corticosteroids, oral antihistamines, oral retinoids, or PUVA [20,21].

Our patient deviated from the classic presentation of PEO as he presented as a healthy, young male with no past medical history of any underlying comorbidities or immunocompromised status. Clinical diagnosis was made after an intensive workup and exclusion of more common differential diagnoses. Thus, our case highlights the importance of considering the possibility of PEO in younger, healthy patients who may not present with the classic predisposing factors. A high index of clinical suspicion and thorough investigation is necessary to prevent misdiagnosis and to rule out likelihood of associated malignancy. Further studies are needed to investigate the causative factors of PEO and to formulate universal guidelines for effective treatment to avoid unnecessary trial and error.

## Conclusions

Papuloerythroderma of Ofuji remains a rare diagnosis with an obscure pathogenesis. Our report presents a patient who lacked the classical background of previously described PEO patients, highlighting the variability in clinical presentation. Clinicians should recognize this entity and its nuances due to its association with internal malignancy and grave consequences of misdiagnosis. Prompt recognition and treatment are generally associated with favorable outcomes in the absence of malignancy.

## References

[1] Ofuji S, Furukawa F, Miyachi Y, Ohno S (1984). Papuloerythroderma. Dermatologica.

[2] Lewis HM, Shahsavari A, Goodman MB (2023). Papuloerythroderma of Ofuji. StatPearls [Internet].

[3] Desai K, Miteva M, Romanelli P (2021). Papuloerythroderma of Ofuji. Clin Dermatol.

[4] Balestri R, Magnano M, Rech G, Zorzi MG, Girardelli CR (2020). Long-term use of methotrexate in Papuloerythroderma of Ofuji. Dermatol Ther.

[5] Allegue F, Fachal C, González-Vilas D, Zulaica A (2018). Papuloerythroderma of Ofuji successfully treated with methotrexate. Dermatol Ther.

[6] Qureshi F, Hughes AJ, Natkunarajah J (2020). Methotrexate for papuloerythroderma of Ofuji. Clin Exp Dermatol.

[7] Terlikowska-Brzósko A, Paluchowska E, Owczarek W, Majewski S (2013). Papuloerythroderma of Ofuji in a 41-year-old woman. Postepy Dermatol Alergol.

[8] Torchia D, Miteva M, Hu S, Cohen C, Romanelli P (2010). Papuloerythroderma 2009: two new cases and systematic review of the worldwide literature 25 years after its identification by Ofuji et al. Dermatology.

[9] Teraki Y, Aso Y, Sato Y (2012). High incidence of internal malignancy in papuloerythroderma of Ofuji: a case series from Japan. Dermatology.

[10] Wang D, Chan MM, Lee HY (2018). Papuloerythroderma of Ofuji presenting as a paraneoplastic phenomenon in myelodysplastic syndrome. Australas J Dermatol.

[11] Lonnee ER, Toonstra J, van der Putte SC, van Weelden H, van Vloten WA (1996). Papuloerythroderma of Ofuji in a HIV-infected patient. Br J Dermatol.

[12] Nomura T, Kodama K, Moriuchi R (2008). Papuloerythroderma of Ofuji associated with early gastric cancer. Int J Dermatol.

[13] Nazzari G, Sabattini C (1999). Ofuji's papuloerythroderma. An association with early gastric cancer. Eur J Dermatol.

[14] Maher AM, Ward CE, Glassman S, Litvinov IV (2018). The importance of excluding cutaneous T-cell lymphomas in patients with a working diagnosis of Papuloerythroderma of Ofuji: a case series. Case Rep Dermatol.

[15] Hur J, Seong JY, Choi TS, Jang JG, Jang MS, Suh KS, Kim ST (2002). Mycosis fungoides presenting as Ofuji's papuloerythroderma. J Eur Acad Dermatol Venereol.

[16] Ueo D, Yoshizumi F, Shirasaka Y (2019). Possible involvement of cancer producing thymic stromal lymphopoietin as an initiator of Papuloerythroderma of Ofuji. Ann Dermatol.

[17] Grob JJ, Collet-Villette AM, Horchowski N, Dufaud M, Prin L, Bonerandi JJ (1989). Ofuji papuloerythroderma. Report of a case with T cell skin lymphoma and discussion of the nature of this disease. J Am Acad Dermatol.

[18] Teraki Y, Inoue Y (2014). Skin-homing Th2/Th22 cells in papuloerythroderma of Ofuji. Dermatology.

[19] Mizuno A, Habe K, Matsushima Y, Kondo M, Yamanaka K (2022). A case of Papuloerythroderma successfully treated with dupilumab. Case Rep Dermatol.

[20] Mufti A, Lytvyn Y, Abduelmula A, Kim P, Sachdeva M, Yeung J (2021). Treatment outcomes in patients with Papuloerythroderma of Ofuji: a systematic review. JAAD Int.

[21] Li S, Yu X, Wang T (2020). Papuloerythroderma of Ofuji. JAMA Dermatol.

